# Impact of prothrombin and factor V Leiden mutations on the progression of fibrosis in patients with chronic hepatitis C

**DOI:** 10.1371/journal.pone.0276592

**Published:** 2022-11-10

**Authors:** Mary Naguib, Wael Abdel-Razek, Suzanne Estaphan, Eman Abdelsameea, Mohamed Abdel-Samiee, Nevine F. Shafik

**Affiliations:** 1 Clinical and Chemical Pathology, National Liver Institute, Menofia University, Shebeen El-Kom, Egypt; 2 Hepatology and Gastroenterology Department, National Liver Institute, Menoufia University, Shebeen El-Kom, Menoufia, Egypt; 3 Physiology Department, Faculty of Medicine, Cairo University, Giza, Egypt; 4 ANU Medical School, Australian National University, Canberra, Australian Capital Territory, Australia; 5 Clinical and Chemical Pathology Department, National Cancer Institute, Cairo University, Giza, Egypt; Karolinska Institutet, SWEDEN

## Abstract

**Background:**

The role of thrombotic factors in the pathogenesis and progression of liver fibrosis remains obscure. We aimed to study the relationship between prothrombin G20210A (PT20210) and factor V Leiden (FVL) mutations and the progression of fibrosis and liver function in chronic HCV patients.

**Methods:**

The study included 100 subjects, 88 patients with HCV-related cirrhosis (compensated: 38, decompensated: 50), and 12 controls. Patients with other viral hepatitis or coinfection, inherited metabolic disease, autoimmune hepatitis, hepatic or extrahepatic malignancy, in addition to patients with causes of hypoalbuminemia, elevated bilirubin or prolonged INR not related to cirrhosis were excluded from the study. Relevant clinical data were collected and basic laboratory tests were performed. Liver fibrosis was assessed using APRI and FIB-4 scores. FVL and PT20210 mutations were analyzed.

**Results:**

FVL and PT20210 mutations were significantly higher in decompensated vs. compensated patients (32% vs. 5.3%, P = 0.001; 20% vs. 5.3%, 0.043, respectively) and absent in controls. Both mutations significantly correlated to the duration of infection, platelet count and fibrosis scores. PT20210 mutation significantly correlated to serum albumin and INR. Both mutations significantly predicted fibrosis scores, especially PT20210 (AUROC: 0.833 for APRI and 0.895 for FIB-4).

**Conclusions:**

Both mutations are significantly correlated to fibrosis progression and liver profile and could be considered as markers predicting the need for early and different intervention.

## 1 Introduction

Hepatitis C virus (HCV) infection is a major worldwide health problem. The highest prevalence of HCV in 2015 was detected in the Eastern Mediterranean region [1, 2]. In Egypt, the Health Issues Survey (HIS) showed a prevalence of HCV seropositivity of 10% in 2015 [3]. However, screening of about 50 million adult citizens, which started in October 2019, revealed that 4.61% had positive HCV antibodies [4].

Chronic hepatitis C occurs in a range of 70 to 80% of cases who contract HCV, which causes damage and progression to cirrhosis within 2–3 decades in 20% of patients; 25% of these patients will develop complications, such as portal hypertension, liver decompensation and hepatocellular carcinoma (HCC), with an average 5-year survival rate of 50% [5].

On the other hand, many HCV-infected patients do not develop liver-related complications even after many years of infection [6]. Therefore, it is essential to study diverse factors that might affect disease progression to early approach the patients with expected complications.

Hypercoagulability is assumed to be a contributing factor for development of liver fibrosis and its progression in chronic liver disease [7]. Thrombin as the end product of coagulation cascade [8] was thought to play a critical role in activating hepatic stellate cells (HSCs) either through binding to protease activated receptor 1 (PAR1) [9] or through induction of activation of TGF- β [10] which is a potent activator of HSCs [11].

Another possible theory depicts formation of occlusive thrombi through thrombin production which results in ischemia, parenchymal destruction ending in liver fibrosis and cirrhosis [12].

FV Leiden (G1691A) and prothrombin (G20210A) mutations are well-known genetic primary hypercoagulable risk factors [13]. Single nucleotide polymorphism of FV Leiden (G1691A) results in amino acid substitution of arginine for glutamine at position 506 of the protein (R506Q), which is one of the cleavage sites of activated protein C (APC). The mutated protein is more resistant to APC cleavage impairing the negative feedback on the coagulation cascade [14].

In addition, it hinders its role in inactivation of FVIIIa [15]. On the other hand, G→A transition in nucleotide 20210 in the 3’-untranslated region leads to an impaired 3’-end cleavage signal, resulting in RNA accumulation and thus, increased prothrombin synthesis [16]. Thus, carriers of these mutations are at high risk of developing venous thromboembolisms [17].

Previous studies have reported the role of thrombotic factors in the development and progression of liver fibrogenesis [18, 19]. However, this role is not clear enough especially with mutations of the thrombotic factors.

Our study aimed to examine the association between prothrombin and factor V Leiden mutations and the progression of fibrosis and liver function in patients infected with HCV.

## 2 Materials and methods

### 2.1. Experimental subjects

This case-control study included 100 subjects between October 2018 and April 2019. A total of 88 adult patients with HCV-related liver cirrhosis were enrolled from the Hepatology and Gastroenterology Department, National Liver Institute, Menoufia University. Cirrhosis was confirmed by clinical examination and radiological findings. Cirrhosis is classified in 2 prognostic stages, compensated and decompensated cirrhosis, based on presence or absence of clinically evident decompensating events especially variceal hemorrhage, ascites and hepatic encephalopathy [HE] [20]. Child-Pugh class A patients were categorized as compensated and Child-Pugh class B or C as decompensated cirrhosis [21]. Duration of infection was assessed in years from the presumed date of infection. Nearly all the patients received tartar emetic injection which was the mass anti-schistosomal treatment in Egypt between 1950s and 80s. The date of tartar emetic injection was presumed as the date of HCV infection [4]. Patients were assessed for antiviral therapy and sample withdrawal was done before starting treatment. Patients with other viral hepatitis coinfection, as hepatitis B virus (HBV), inherited metabolic disease, autoimmune hepatitis, or hepatic or extrahepatic malignancy were excluded from the study. Patients with causes of hypoalbuminemia (nephrotic syndrome or diabetic nephropathy), elevated bilirubin (hemolysis or biliary obstruction) or prolonged INR (use of anticoagulants) not related to cirrhosis were also excluded. Twelve apparently healthy subjects were included as controls from the blood donation unit of National Liver Institute, Menoufia University. They had normal liver tests and were seronegative for viral hepatitis B and C.

This study was approved by the local institutional review board. Written informed consents were obtained from all eligible subjects before they were enrolled into the study. The study conforms to The Code of Ethics of the World Medical Association (Declaration of Helsinki), printed in the British Medical Journal (18 July 1964).

### 2.2. Routine laboratory investigations

All the included subjects had a thorough history taking, clinical examination and abdominal ultrasonography. Blood samples were obtained for complete blood count [Sysmex XT-1800i (Sysmex Corporation, Kobe, Japan)], liver and renal tests, anti-HCV, HB surface Ag (HBsAg), anti HB core IgG (anti-HBc IgG) and alpha-fetoprotein [Cobas 6000 (Roche Diagnostics GmbH, Mannheim, Germany)]. Prothrombin time and international normalized ration (INR) were assessed using Sysmex CS-1600 (Sysmex Europe GmbH, Norderstedt, Germany).

### 2.3. Evaluation of fibrosis

Stage of fibrosis was assessed noninvasively using the FIB-4 [22] and aspartate aminotransferase (AST) to platelet ratio index (APRI) [23] scores as follows:

-
$FiB-4=\;\frac{Age\;\left(years\right)\times AST\;\left(IU/l\right)}{Platelet\;count\;\left(x10^{9}/l\right)x\sqrt{ALT\;\left(IU/l\right)}}$

-
$APRI=\frac{AST\;\left(IU/l\right)/AST\;upper\;limit\;of\;normal\;(IU/l)}{Platelet\;count\;\left(10^{9}/l\right)}$

### 2.4. Studying factor V Leiden (G1691A) and prothrombin (G20210A) mutation

The genomic DNA was isolated from the peripheral blood of all subjects using QIAamp DNA blood Mini Kit (Qiagen, Hilden, Germany) following standard procedures according to the manufacturer’s instructions. Genomic DNA was examined for quality and quantity using the Nano Drop^®^-1000 spectrophotometer (Nanodrop Technologies, Inc., Wilmington, NC).

Analyses of gene mutations of FVL and PT20210 were performed using hydrolysis probes via snpsig real-time PCR mutation detection/ allelic discrimination kit (Applied Biosystems Inc., CA, USA), in a lightCycler instrument (Roche Diagnostics, Mannheim, Germany). Genotyping reaction mix included 20 ng of whole blood genomic extracted DNA and the following reagents: 1 μL of primer / probe mix (Genotyping primer/probe mix contains two labelled probes homologous to the wild and mutant genotypes under investigation.), 10 μL Taqman universal master mix II No UNG (Catalog no. 4440040), and complete volume to 25 μL using RNAse/DNase free water. A negative control was included in each reaction to exclude DNA contamination. The thermal cycling profile was 2 min at 95°C for enzyme activation followed by 10 cycles of 10 s DNA denaturation at 95°C, 60 s extension at 60°C, followed by 50 cycles of 10 s DNA denaturation at 95°C, 60 s extension at 66°C where the fluorogenic data were collected through the ROX and VIC channels.

### 2.5. Statistical methods

Controls and patients with compensated and decompensated cirrhosis were compared using ANOVA then post-hoc Tukey’s test for intergroup comparisons regarding quantitative variables and Chi-square test for categorical variables. Spearman’s non-parametric correlations between FVL and PT20210 mutations and other variables were assessed. The ability of FVL and PT20210 mutations to predict fibrosis as assessed by FIB-4 and APRI was tested with the receiver-operating characteristics (ROC) curve and area under the ROC curve (AUROC).

Data were analyzed using IBM SPSS Statistics for Macintosh, Version 22.0 (IBM Corp, Armonk, NY, USA). All analyses were two-sided and P less than 0.05 was considered statistically significant.

## 3 Results

### 3.1. Characteristics of hepatitis C cirrhotic patients

This study included 88 patients with HCV-related liver cirrhosis, 38 (43.2%) with compensated, 50 (56.8%) with decompensated cirrhosis, and 12 controls. Controls and patients with compensated and decompensated cirrhosis had similar gender distribution as they were mostly males (66.7%, 57.9%, 64% respectively, P = 0.792). Controls had significantly younger age than both the compensated and decompensated groups (48 ± 4.1, 56.3 ± 6.5, 59.6 ± 7.6 years, respectively). Patients with compensated and decompensated cirrhosis were comparable regarding age (Table 1).

**Table 1:** Comparison of patients and controls.

		Controls	Compensated	Decompensated	P
Age (years)		48 ± 4.1	56.3 ± 6.5	59.6 ± 7.6	<0.0001^a^^,^^b^
AST (IU/L)		24.8 ± 5.1	52.6 ± 20.9	76.1 ± 52.2	<0.0001^b^^,^^c^
ALT (IU/L)		26.2 ± 3.3	48.6 ± 22.1	48.1 ± 40.8	0.085
GGT (IU/L)		38 ± 25.9	67.9 ± 27.4	92.8 ± 94.7	0.035^b^
Alkaline phosphatase (IU/L)		58.5 ± 38.6	86.3 ± 27.3	140.5 ± 103.6	<0.0001^b^^,^^c^
Total bilirubin (mg/dl)		0.9 ± 0.3	1 ± 0.3	5.4 ± 4.6	<0.0001^b^^,^^c^
Albumin (g/dl)		4.8 ± 0.1	3.9 ± 0.5	2.2 ± 0.4	<0.0001^a^^,^^b^^,^^c^
Urea (mg/dl)		34.8 ± 3.1	67.8 ± 46	104.7 ± 60.6	<0.0001^b^^,^^c^
Creatinine (mg/dl)		0.8 ± 0.1	1 ± 0.2	1.8 ± 1	<0.0001^b^^,^^c^
Prothrombin concentration (%)		97.2 ± 1	77.2 ± 9.3	41.9 ± 8.8	<0.0001^a^^,^^b^^,^^c^
INR		1 ± 0.01	1.2 ± 0.1	1.9 ± 0.6	<0.0001^b^^,^^c^
Hemoglobin (g/dl)		13.4 ± 1.9	11.6 ± 2	10 ± 1.2	<0.0001^a^^,^^b^^,^^c^
WBCs (x10^3^/mm^3^)		7.6 ± 0.9	6.8 ± 1.8	4.6 ± 1.9	<0.0001^b^^,^^c^
Platelets (x10^3^/mm^3^)		292.5 ± 23.2	176.9 ± 17.5	103.2 ± 41.2	<0.0001^a^^,^^b^^,^^c^
APRI		0.2 ± 0.1	0.8 ± 0.3	2.1 ± 1.5	<0.0001^b^^,^^c^
FIB-4		0.8 ± 0.2	2.5 ± 0.9	7.8 ± 4.2	<0.0001^b^^,^^c^
Duration of infection (years)		-	33.8 ± 7.1	29.3 ± 7.9	0.007^c^
Gender*	Males	8 (66.7)	22 (57.9)	32 (64)	0.792
	Females	4 (33.3)	16 (42.1)	18 (36)	
Factor VL polymorphism*	Wild	12 (100)	36 (94.7)	34 (68)	0.001
	Mutant	0	2 (5.3)	16 (32)	
Factor II polymorphism*	Wild	12 (100)	36 (94.7)	40 (80)	0.043
	Mutant	0	2 (5.3)	10 (20)	

Data are n (%), otherwise, mean ± standard deviation.

P<0.05 when comparing

^a^ controls and patients with compensated cirrhosis,

^b^ controls and patients with decompensated cirrhosis,

^c^ patients with compensated and decompensated cirrhosis.

ALT, alanine aminotransferase; APRI, aspartate aminotransferase to platelet ratio index; AST, aspartate aminotransferase; GGT, gamma-glutamyl transferase; INR, international normalized ratio; WBCs, white blood cells.

When compared to patients with compensated cirrhosis, those with decompensated cirrhosis had significantly higher AST, alkaline phosphatase, serum total bilirubin, urea and creatinine, and INR, and more advanced fibrosis as reflected by the APRI and FIB-4 scores. They also had significantly shorter duration of infection, lower prothrombin concentration, albumin, hemoglobin, white blood cell and platelet count (Table 1).

### 3.2. Prevalence of factor V Leiden (G1691A) and prothrombin G20210A polymorphisms

Both mutations could not be detected in the control subjects. In the studied patients with cirrhosis, the prevalence of FVL and PT20210 mutant polymorphisms was 20.5% and 13.6% respectively. The difference between patients and controls in the distribution of FVL or PT20210 mutations failed to reach statistical significance (P = 0.576 and 1.000, respectively). No homozygous mutations were detected for FVL or PT20210 mutation.

The mutant forms of FVL and PT20210 polymorphism were significantly higher in the decompensated group compared to the compensated group (P = 0.001and 0.043, respectively, Table 1).

### 3.3. Associations of factor V Leiden and prothrombin G20210A polymorphisms

Both FVL and PT20210 polymorphisms had significant positive correlation with APRI score (P = 0.049 and 0.004, respectively) and FIB-4 (P = 0.027 and 0.001, respectively), and negative correlation with platelet count (P = 0.036 and 0.001, respectively) and duration of infection (P = 0.008 and 0.010 respectively). PT20210 polymorphism was significantly positively correlated with INR (P = 0.016) and significantly negatively correlated with GGT, prothrombin concentration and serum albumin (P = 0.030, 0.025 and 0.003, respectively) (Table 2).

**Table 2:** Correlations of factors II and V polymorphisms.

	Factor V polymorphism		Factor II polymorphism	
	Coefficient	P	Coefficient	P
Gender	-0.152	0.291	0.091	0.528
Age (years)	0.251	0.078	0.197	0.171
AST (IU/l)	0.200	0.163	0.158	0.274
ALT (IU/l)	-0.043	0.765	-0.049	0.735
GGT (IU/l)	0.092	0.525	-0.307	0.030
Alkaline phosphatase (IU/l)	0.108	0.454	-0.043	0.769
Total bilirubin (mg/dl)	0.208	0.116	0.145	0.353
Albumin (g/dl)	-0.209	0.144	-0.408	0.003
Urea (mg/dl)	0.103	0.477	0.092	0.526
Creatinine (mg/dl)	0.054	0.708	0.013	0.930
Prothrombin concentration (%)	-0.231	0.107	-0.316	0.025
INR	0.258	0.070	0.339	0.016
Duration of infection (years)	-0.392	0.008	-0.385	0.010
Hemoglobin (g/dl)	-0.125	0.388	-0.224	0.117
WBCs (x103/mm3)	0.007	0.960	-0.111	0.443
Platelets (x103/mm3)	-0.298	0.036	-0.444	0.001
APRI	0.280	0.049	0.401	0.004
FIB-4	0.312	0.027	0.461	0.001

ALT, alanine aminotransferase; APRI, aspartate aminotransferase to platelet ratio index; AST, aspartate aminotransferase; GGT, gamma-glutamyl transferase; INR, international normalized ratio; WBCs, white blood cells

### 3.4. Prediction of fibrosis in patients using factor V and factor II polymorphisms

**Fig 1:**
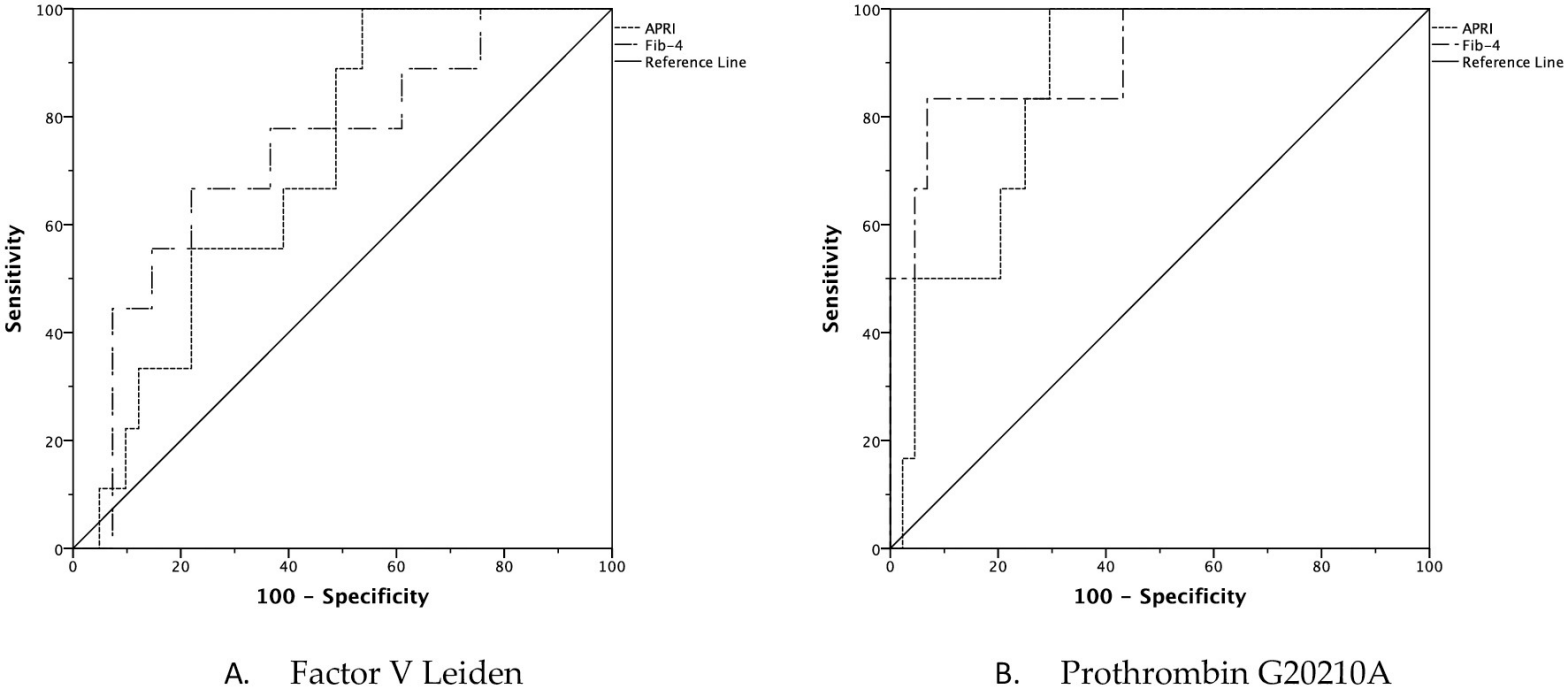
Prediction of fibrosis in patients using factor V Leiden and prothrombin 20210 polymorphisms.

The ROC curve was used to assess the ability of FVL and PT20210 polymorphisms to predict FIB-4 and APRI scores, non-invasive indices of fibrosis, in patients with HCV-related cirrhosis. Both polymorphisms predicted significantly FIB-4 and APRI scores. However, the polymorphism of PT20210 had a higher AUROC when compared to that of FVL in prediction of both APRI (0.833 vs. 0.660, respectively) and FIB-4 (0.895 vs. was 0.689, respectively) (Table 3 and Fig 1).

**Table 3:** Area under the curve of factor V Leiden and prothrombin polymorphisms in prediction of fibrosis.

		Area under the curve	95% CI	P
Factor V Leiden	APRI	0.660	0.536–0.784	0.037
	FIB-4	0.689	0.543–0.835	0.014
Prothrombin polymorphism	APRI	0.833	0.739–0.928	<0.0001
	FIB-4	0.895	0.788–1.000	<0.0001

APRI, aspartate aminotransferase to platelet ratio index; CI, confidence interval.

## 4 Discussion

Chronic infection with HCV has been an extensive health problem in Egypt [24]. Liver fibrosis is an inevitable consequence of chronic hepatitis C, which may result in development of liver cirrhosis and portal hypertension if left untreated [25].

With the revolutionary treatment of HCV with oral direct acting antivirals (DAAs), high cure rates in different clinical settings are achieved, including elderly patients, end-stage renal disease patients and those with cirrhosis [26]. However, still several disputes remain including hard to- cure cirrhosis [27].

Thus studying different factors that affect progression of fibrosis could help to identify high risk patients for development of complication of fibrosis to be recruited in early antiviral treatment and continually followed up. Also, it might provide data for development of new therapies for those patients.

Thrombosis might play an influential role in the progression of liver fibrosis [28]. Thrombin not only leads to thrombosis, but also activates liver stellate cells and promotes fibrogenesis through activation of fibrogenic factors such as transforming growth factor TGF-β. Also, ischemia associated with local thrombosis of portal sinusoids up-regulates the expression and secretion of profibrogenic factors including platelet derived growth factor (PDGF). FVL polymorphism prevents normal anti-thrombin activity of activated protein C and can accelerate the fibrogenetic process [29].

Therefore, we aimed to study the potential effect of PT20210 and FVL mutations on progression of fibrosis and liver function.

The FVL and PT20210 mutant polymorphisms could not be detected in the control subjects. Moreover, no homozygous mutations could be detected for FVL or PT20210 mutation in the studied controls or patients. These findings are similar to previous reports studying thrombophilic tendency among Egyptian population. Both Yaman et al [30] and El Baz et al [31] couldn`t detect FVL mutation in control and Fawzy et al [32] and Essa et al [33] reported no PT 20210 mutation in control. Jadaon [34] stated that Prothrombin G20210A mutation was found to be very rare or even absent in Asian and African populations. Besides, Yaman et al [30] and Fawzy et al [32] could not detect homozygous mutation of FVL in patient or control groups, however, El Baz et al [31] and Essa et al [33], described homozygosity of both FVL G1691A and PT 20210 in patient groups. These differences could be due to small sample sizes and studying different presentations of thrombophilic tendency.

There was significant positive correlation between FVL and PT20210 mutations and the non-invasive markers of fibrosis, APRI and FIB-4. Previous studies reported increased fibrosis progression with both mutations [35, 36]. Wright et al. reported the significant correlation between FVL mutation, specifically, and the rate of progression of fibrosis in chronic HCV patients [36]. However, they did not find significant association between prothrombin mutation and rate of fibrosis. Poujol-Robert et al. [37] highlighted the importance of APC resistance as an independent risk factor for cirrhosis progression. On the other hand, Maharshak et al. found that the presence of the PT20210 mutation caused fast progression of liver fibrosis [38], however, they could not detect significant association between FVL mutation and rate of fibrosis progression, which they explained could be due to their relatively small sample size [38].

In contrast, Dik et al. [28] could not detect correlation between PT20210 or FVL mutations and fibrosis progression rate. This could be due to differences in studied population. They noticed that highest fibrosis progression rate was in Caucasian and Black patients than in Asians. Also they reported that rate of fibrosis progression was higher in chronic HCV patients than chronic HBV patients. However, they stated that factor XIII Val34Leu mutation was a risk factor for an increased rate of liver fibrogenesis in patients with chronic hepatitis B or C infection [28]. Goulding et al., [39] denied the impact of FVL or PT20210 polymorphisms on fibrosis scores in HCV-infected patients as well. This could be due to the low overall fibrosis stage in their study making it difficult to observe any difference. In addition, the mean interval time of his study was too short to definitively assess the rate of fibrosis progression.

Both mutations were significantly correlated to the platelet count which was significantly lower in patients with decompensated cirrhosis compared to the compensated group. Together with the significantly higher prevalence of both mutations in the decompensated vs. compensated groups, we could deduce that FVL and PT20210 mutations are associated with the portal pressure, for which the platelet count is an indirect marker, and which is another complication of liver fibrosis.

Interestingly, patients with decompensated cirrhosis had a significantly shorter presumed duration of infection and higher prevalence of both mutations when compared to those to compensated cirrhosis. These findings could imply that patients with decompensated cirrhosis are rapid fibrosers. This could be partly explained by the higher prevalence of the mutations of these thrombotic factors.

We further studied the effect of both mutations on the synthetic function of the liver. We noticed that PT20210 and FVL were significantly associated with liver decompensation. PT20210 mutation significantly correlated to synthetic functions of the liver, as reflected by serum albumin, prothrombin concentration and INR. Both mutations correlated to platelet count and duration of infection. Previously, Goulding et al. [39] detected significantly lower ALT levels for factor V wild type compared to heterozygotes. However, to the best of our knowledge, this is the first study to assess the correlation of coagulation factor mutations with the progression to decompensation. Clinical implication of our findings is strict follow up of patients with cirrhosis who have such mutations due to high risk of developing complications and also it will be beneficial to know their potential role in the pathogenesis of fibrosis that may be a target of therapy in the future. Screening of hepatocellular carcinoma in rapid fibrosers with such mutation may be warranted.

Limitations of our study include that it was a single center study, which included only one etiology of liver disease, HCV-related liver cirrhosis, and with no prospective follow-up.

## 5 Conclusions

Our study has demonstrated that factor V Leiden (G1691A) and prothrombin G20210 mutant polymorphisms are significantly correlated with the liver fibrosis, synthetic function. Notably, both mutations, especially PT20210 significantly predicted liver fibrosis. This could reflect the role of thrombotic mutations in the pathogenesis of fibrosis and could be the target of future antifibrotic therapies. Further longitudinal studies are recommended.
